# Supplementary material for: Dielectrocapillarity for exquisite control of fluids
Source: Nat Commun. 2026 Feb 12;17:2661. doi: 10.1038/s41467-026-69482-1 (PMC13009173; doi:10.1038/s41467-026-69482-1)
Supplement: Supplementary file 1 — Supplementary Information [file 41467_2026_69482_MOESM1_ESM.pdf]

CONTENTS

S1. Theory S2
S1.1. First principles theory for electromechanics in liquids S2
S1.2. Putting theory into practice S2

S2. Molecular model for a polar fluid S3

S3. Validation of the theory S4
S3.1. Homogeneous bulk system S4
S3.2. Inhomogeneous system S7

S4. Generation of training data S7

S5. Training S9

S6. Using the neural functionals S11

S7. Additional results that support the main article S12
S7.1. Bulk equations of state and liquid–vapor coexistence with no external electric field S12
S7.2. Bulk response and phase behavior under spatially-varying external electric fields S14
S7.3. Water S18
S7.4. Interdigitated electrode simulations S19

References S22

S1. THEORY

S1.1. First principles theory for electromechanics in liquids

Here, we will state the key results of the recently formulated liquid state theory capable of describing electromechanics as an emergent phenomenon. For detailed derivations and discussions, we refer the reader to Ref. 1. For an inhomogeneous single-component fluid in the grand canonical ensemble, its full electromechanical response can be exactly derived from the grand potential functional,

$$\Omega([\varrho, \beta\varphi], T) = \mathcal{F}^{(\mathrm{id})}_{\mathrm{intr}}([\varrho], T) + \mathcal{F}^{(\mathrm{ex})}_{\mathrm{intr}}([\varrho, \beta\varphi], T) + \int \mathrm{d}\boldsymbol{r}\, \varrho(\boldsymbol{r})[V_{\mathrm{ext}}(\boldsymbol{r}) - \mu], \tag{S1}$$

where the ideal intrinsic Helmholtz free energy is $\mathcal{F}^{(\mathrm{id})}_{\mathrm{intr}}([\varrho], T) = k_{\mathrm{B}}T \int \mathrm{d}\boldsymbol{r}\, \varrho(\boldsymbol{r})[\ln \zeta^{-1}\Lambda^3 \varrho(\boldsymbol{r}) - 1]$, with Λ denoting the thermal de Broglie wavelength, ζ a partition function accounting for intramolecular molecular degrees of freedom of each fluid particle, and T the temperature (k_{B} is the Boltzmann constant). The non-electrostatic external potential is V_{ext}, and the chemical potential is μ. The excess part of the intrinsic Helmholtz free energy functional $\mathcal{F}^{(\mathrm{ex})}_{\mathrm{intr}}$ has the usual functional dependence on the number density field, $\varrho(\boldsymbol{r})$. In addition, it also has a functional dependence on the thermally scaled electrostatic potential $\beta\varphi$, where $\beta = 1/k_{\mathrm{B}}T$. Since we are interested in phase behavior, we also indicate the parametric dependence on T explicitly, as done in Ref. 2.

To solve for the full electromechanical response of the fluid exactly, one must know the form of $\mathcal{F}^{(\mathrm{ex})}_{\mathrm{intr}}([\varrho, \beta\varphi], T)$, which is a highly complex object due to the many-body correlations in the fluid. Through the development of neural functional techniques,[3] such a free energy functional is accessible by deep learning from grand canonical simulation data. Specifically, one can represent its first functional derivatives with a neural network, while free energies are accessible by functional line integration. Moreover, higher-order correlations are also accessible by autodifferentiation. In our case, the first functional derivatives give the one-body direct correlation functional,

$$c^{(1)}(\boldsymbol{r}; [\varrho, \beta\varphi], T) = -\frac{\delta \beta \mathcal{F}^{(\mathrm{ex})}_{\mathrm{intr}}([\varrho, \beta\varphi], T)}{\delta \varrho(\boldsymbol{r})}, \tag{S2}$$

and the one-body charge density functional,

$$n^{(1)}(\boldsymbol{r}; [\varrho, \beta\varphi], T) = \frac{\delta \mathcal{F}^{(\mathrm{ex})}_{\mathrm{intr}}([\varrho, \beta\varphi], T)}{\delta \beta\varphi(\boldsymbol{r})}. \tag{S3}$$

The grand potential functional follows the variational principle of standard classical density functional theory,[4,5]

$$\left.\frac{\delta \Omega([\varrho, \beta\phi], T)}{\delta \varrho(\boldsymbol{r})}\right|_{\varrho=\rho} = 0, \tag{S4}$$

and its generalization for complex observables from hyperdensity functional theory.[6] Therefore, under an applied electrostatic potential $\beta\phi(\boldsymbol{r})$, the corresponding number density $\rho(\boldsymbol{r})$ at equilibrium is given exactly by the Euler–Lagrange equation,

$$\rho(\boldsymbol{r}) = \frac{\zeta}{\Lambda^3} \exp\left(-\beta V_{\mathrm{ext}}(\boldsymbol{r}) + \beta\mu + c^{(1)}(\boldsymbol{r}; [\rho, \beta\phi], T)\right). \tag{S5}$$

The equilibrium charge density $n(\boldsymbol{r})$ is also given by

$$n(\boldsymbol{r}) = n^{(1)}(\boldsymbol{r}; [\rho, \beta\phi], T). \tag{S6}$$

S1.2. Putting theory into practice

A key condition for the success of neural functional techniques is the locality of the functional mappings. However, for fluids whose intermolecular interactions are long-ranged, such as ionic and dielectric fluids, so too are their molecular correlations. Consequently, the functional dependencies of $c^{(1)}(\boldsymbol{r}; [\varrho, \beta\varphi], T)$ and

$n^{(1)}(\boldsymbol{r};[\varrho,\beta\varphi],T))$ are, in general, non-local. In Ref. 1, we show that one strategy to overcome this obstacle is to split the excess free energy functional,

$$\mathcal{F}_{\mathrm{intr}}^{(\mathrm{ex})}([\varrho,\beta\varphi],T)=\mathcal{F}_{\mathrm{intr,R}}^{(\mathrm{ex})}([\varrho,\beta\varphi],T)+\Delta\mathcal{F}_{\mathrm{intr}}^{(\mathrm{ex})}([\varrho,\beta\varphi],T),\tag{S7}$$

where $\mathcal{F}_{\mathrm{intr,R}}^{(\mathrm{ex})}$ defines the excess free energy functional of a reference system whose electrostatic interactions are completely short-ranged. Quantities pertaining to this reference system are indicated with the "R" subscript. In this work, we split the Coulomb potential,

$$\frac{1}{r}=v_0(r)+v_1(r),\tag{S8}$$

with $v_0=\mathrm{erfc}(\kappa r)/r$ and $v_1=\mathrm{erf}(\kappa r)/r$ (i.e., κ^{-1} defines a range separation), and the reference system's electrostatic interactions are presribed by v_0. Due to the now short-ranged nature of the interaction potential, such a reference system's free energy functional $\mathcal{F}_{\mathrm{intr,R}}^{(\mathrm{ex})}$ is, by construction, local. This local nature of the reference system makes it amenable for the local learning strategy of neural functional theory. Provided that κ^{-1} is chosen large enough, any remaining non-local contributions (now contained in the functonal $\Delta\mathcal{F}_{\mathrm{intr}}^{(\mathrm{ex})}$) can be approximated with high accuracy with a mean field form,

$$\Delta\mathcal{F}_{\mathrm{intr}}^{(\mathrm{ex})}([\varrho,\beta\varphi],T)=\Delta\mu\int\mathrm{d}\boldsymbol{r}\,\varrho(\boldsymbol{r})\;+\frac{1}{2}\int\mathrm{d}\boldsymbol{r}\int\mathrm{d}\boldsymbol{r}'\,n_{\mathrm{R}}^{(1)}(\boldsymbol{r};[\varrho,\beta\varphi],T)n_{\mathrm{R}}^{(1)}(\boldsymbol{r}';[\varrho,\beta\varphi],T)v_1(|\boldsymbol{r}-\boldsymbol{r}'|).\tag{S9}$$

This usefulness of this principle of a well-controlled mean field approximation for electrostatic interactions has been demonstrated extensively in the context of local molecular field theory applied to computer simulations of ions and water.[7–14] Recently, we also applied it in a density functional setting to ionic fluids,[15] in which we employed molecular models where the relationship between the charge and number densities is trivial. In this paper, we will also use it in a density functional context for a polar fluid. This is a much more challenging problem, as the relationship between $n^{(1)}(\boldsymbol{r},[\varrho,\beta\varphi],T)$ and ϱ is unknown.

Following Ref. 1, the first functional derivatives of the excess free energy can be given by

$$c^{(1)}(\boldsymbol{r};[\rho,\beta\phi],T)=c_{\mathrm{R}}^{(1)}(\boldsymbol{r};[\rho,\beta\phi_{\mathrm{R}}],T)-\beta\Delta\mu,\tag{S10}$$

$$n^{(1)}(\boldsymbol{r};[\rho,\beta\phi],T)=n_{\mathrm{R}}^{(1)}(\boldsymbol{r};[\rho,\beta\phi_{\mathrm{R}}],T),\tag{S11}$$

where

$$\phi_{\mathrm{R}}(\boldsymbol{r})=\phi(\boldsymbol{r})+\int\mathrm{d}\boldsymbol{r}'\,n_{\mathrm{R}}^{(1)}(\boldsymbol{r}';[\rho,\beta\phi],T)\,v_1(|\boldsymbol{r}-\boldsymbol{r}'|).\tag{S12}$$

The potential ϕ_{R} (dubbed the "restructuring electrostatic potential"), accounts for the average effects of long-ranged electrostatics that have been omitted in the short-ranged reference system. Finally, $\Delta\mu$ can be directly related to bulk compressibilities of the fluid (see Sec. S3).

To summarize the practical implementation of the theory:

- We learn the local functionals $c_{\mathrm{R}}^{(1)}(\boldsymbol{r};[\varrho,\beta\varphi],T)$ and $n_{\mathrm{R}}^{(1)}(\boldsymbol{r};[\varrho,\beta\varphi],T)$ from simulation data of a short-ranged reference polar fluid via the neural functional method.
- At a specified μ, T, $V_{\mathrm{ext}}(\boldsymbol{r})$ and $\phi(\boldsymbol{r})$, we numerically solve the set of equations S5, S6, S10, S11 and S12 to obtain the equilibrium structure, i.e., the number density $\rho(\boldsymbol{r})$ and charge density $n(\boldsymbol{r})$ of the long-ranged polar fluid of interest.
- Thermodynamic information about the system is available via $\Omega([\rho,\phi],T)$ by functional calculus, including the phase behavior that we explore in this article.

S2. MOLECULAR MODEL FOR A POLAR FLUID

We consider a Stockmayer-like model[16] for the dipolar fluid, in which the dipole is represented by point charges separated by a fixed distance. Specifically, each fluid particle is a rigid linear triatomic molecule, with

equal and opposite charges $q_+ = -q_-$ located on the "end-atoms" (B and C) with a Lennard–Jones center (A) located halfway along the separation vector of B and C. The distance $d_{\rm BC}$ between the point charges is fixed. The potential energy function of the full system takes the form

$$\mathcal{U}(\boldsymbol{R}^N) = \sum_{i,i<j}^{N} u_{\rm LJTS}(|\boldsymbol{r}_{{\rm A},j} - \boldsymbol{r}_{{\rm A},i}|) + \sum_{i,i<j}^{N}\sum_{\alpha,\gamma} \frac{q_{\alpha,i}q_{\gamma,j}}{|\boldsymbol{r}_{\alpha,j} - \boldsymbol{r}_{\gamma,i}|}, \tag{S13}$$

where $\boldsymbol{R}^N$ denotes the set of atomic positions for a configuration of N dipolar molecules, while $\boldsymbol{r}_{\alpha,i}$ indicates the position of site α on molecule i. For equations involving electrostatic interactions, we adopt a unit system in which $4\pi\epsilon_0 = 1$, where ϵ_0 is the permittivity of free space. Non-electrostatic interactions between molecules are modelled with the truncated and shifted Lennard–Jones potential,

$$u_{\rm LJTS}(r) = \begin{cases} u_{\rm LJ}(r) - u_{\rm LJ}(r_{\rm c}) & r \le r_{\rm c} \\ 0 & r > r_{\rm c}, \end{cases} \tag{S14}$$

with

$$u_{\rm LJ}(r) = 4\varepsilon_{\rm LJ}\left[\left(\frac{\sigma_{\rm LJ}}{r}\right)^{12} - \left(\frac{\sigma_{\rm LJ}}{r}\right)^{6}\right], \tag{S15}$$

parameterized by an energy scale $\varepsilon_{\rm LJ}$, a length scale $\sigma_{\rm LJ}$ and a cut-off distance $r_{\rm c}$. The potential energy function of the short-ranged reference system takes the form

$$\mathcal{U}_{\rm R}(\boldsymbol{R}^N) = \sum_{i,i<j}^{N} u_{\rm LJTS}(|\boldsymbol{r}_{{\rm A},j} - \boldsymbol{r}_{{\rm A},i}|) + \sum_{i,i<j}^{N}\sum_{\alpha,\gamma} q_{\alpha,i}q_{\gamma,j}v_0(|\boldsymbol{r}_{\alpha,j} - \boldsymbol{r}_{\gamma,i}|). \tag{S16}$$

For the dipolar fluid, we employ the following parameters,[17] $\sigma_{\rm LJ} = 3.024\,$Å, $\varepsilon_{\rm LJ} = 1.87\,{\rm kJ\,mol^{-1}}$, $|q_+| = |q_-| = 0.382\,e$ with e being the elementary charge, $d_{\rm BC} = 1\,$Å such that the molecular dipole moment is $p = q_+d_{\rm BC} = 1.835\,$D, $r_{\rm c} = 10\,$Å. These parameters give a fluid that has a density and dielectric constant similar to those of liquid water at room temperature.[18] For the short-ranged reference system, we use $\kappa^{-1} = 4.5\,$Å (see Sec. S3). In the main paper, we report quantities in their reduced units: $T^* = k_{\rm B}T/\varepsilon_{\rm LJ}$, $p^* = (p^2/\varepsilon_{\rm LJ}\sigma_{\rm LJ}^3)^{1/2} = 2$, $\rho^* = \rho\sigma_{\rm LJ}^3$ and $E^* = E\sigma^{3/2}\varepsilon_{\rm LJ}^{-1/2}$. In this supporting information, to stay in line with how the simulations and cDFT calculations are performed in practice, we will report quantities in "real" units. For water, we used the SPC/E model[19] which has the following parameters $\sigma_{\rm LJ} = 3.166\,$Å, $\varepsilon_{\rm LJ} = 0.650\,{\rm kJ\,mol^{-1}}$, $p = 2.351\,$D and $r_{\rm c} = 10\,$Å.

For comparison to electrophoretic rise in ionic fluids, we employ cDFT as developed in Ref. 15 for the restricted primitive model (RPM). The fluid is at a supercritical temperature, $T^* = 0.066$. The hard sphere diameter is $\sigma = 2.76\,$Å, with charge $|q_+| = |q_-| = e$, corresponding to molten NaCl at $T = 4000\,$K. For the result in Fig. 1b in the main paper, $E_{\rm max} = 2\,{\rm V\,Å^{-1}}$ for the RPM.

S3. VALIDATION OF THE THEORY

Before introducing the ML/cDFT calculations, we will demonstrate that: (i) $\kappa^{-1} = 4.5\,$Å is a sufficient choice for the short-ranged reference system; and (ii) the mean-field expression for the electrostatic restructuring potential and thermodynamic corrections for long-ranged electrostatics are both highly accurate and give thermodynamic consistency. We will use computer simulations to do this, focusing on the dipolar fluid. We note that previous, simulation-based, local molecular field treatments of SPC/E water have validated the choice of $\kappa^{-1} = 4.5\,$Å.[7,20]

S3.1. Homogeneous bulk system

When κ^{-1} is sufficiently large, the difference in bulk free energies between the LR and SR systems will be dominated by differences in the potential energy. In Ref. 20, an analytical correction for the average Coulombic energy between the long-ranged and short-ranged bulk systems, based on the Stillinger–Lovett

moment conditions,[21] is derived. Specifically, for a neutral polar fluid of N molecules,

$$\Delta U \equiv U - U_R = \frac{N}{2\beta\rho_b\kappa^{-3}\sqrt{\pi}^3}\frac{\epsilon-1}{\epsilon} - \frac{2Np^2}{3\kappa^{-3}\sqrt{\pi}}, \tag{S17}$$

where ρ_b is the average bulk density, and ϵ is the dielectric constant of the fluid. Since ϵ is an intensive material property that is determined by short-ranged correlations, a well-chosen SR reference should have the same ϵ as the LR system. Moreover, in Ref. 1, we show that this could further be verified by considering the charge–charge response function $\hat{\chi}^{(n)}_{n,\mathrm{R}}(k)$ of the SR system,

$$\lim_{k\to 0}\frac{4\pi\beta}{k^2}\hat{\chi}^{(n)}_{n,\mathrm{R}}(k) = \frac{(\epsilon-1)/\epsilon}{1-\left(\frac{\epsilon-1}{\epsilon}\right)\exp(-\frac{k^2}{4\kappa^2})} = \epsilon - 1. \tag{S18}$$

We demonstrate that this is true in Fig. S1.

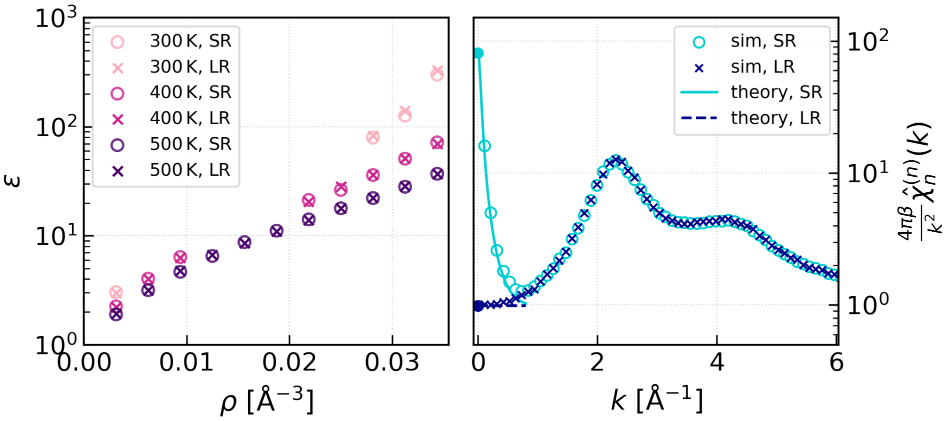

Figure S1: Consistency of dielectric response between SR and LR systems. (a) The dielectric constant is the same for the homogeneous SR and LR systems, as computed using the polarization fluctuations at zero field[22,23] from MD simulations at different densities and temperatures. (b) The charge–charge response functions from MD simulations show agreement with the theory: while $4\pi\beta\chi^{(n)}_n/k^2$ of the LR fluid tends to $(\epsilon-1)/\epsilon$, that of the SR tends to $\epsilon - 1$ according to Eq. S18. The result is shown representatively for $T = 300$ K, $\rho_b = 0.028$ Å^{-3}. Even though the SR and LR systems disagree, we emphasize that the same value $\epsilon = 82$ determines the $k \to 0$ limit in both cases.

Having established that ϵ is consistent between the SR and LR systems, we verified that the total potential energy of the LR system can be reproduced with ΔU given by Eq. S17 for any choice of $\kappa^{-1} > 3$ Å. This is shown in Fig. S2(a). We also show in Fig. S2(b) that, with $\kappa^{-1} = 4.5$ Å^{-1}, the reference system can faithfully capture the short-ranged local structure of the true system, reflected in the bulk radial distribution functions.

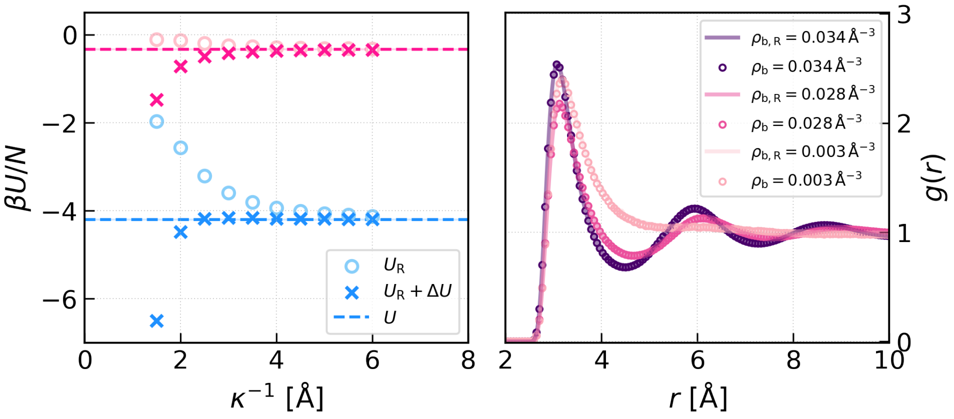

Figure S2: Consistency in bulk energy and structure between SR and LR systems. (a) The total potential energy of the reference SR system at various κ^{-1} without (circles) and with the long-range correction (crosses) as given by Eq. S17. The energies (at different densities) of the full LR system are indicated by the horizontal dashed lines. Results are shown representatively for $T = 500\,\text{K}$, $\rho_\text{b} = 0.028\,\text{Å}^{-3}$ (blue) and $T = 500\,\text{K}$, $\rho_\text{b} = 0.0063\,\text{Å}^{-3}$ (pink). (b) With $\kappa^{-1} = 4.5\,\text{Å}^{-1}$, the radial distribution functions of the SR system (lines) are in good agreement with the LR system (dots), shown representatively for different densities at $T = 500\,\text{K}$.

From the expression for ΔU given in Eq. S17, corrections for both the pressure[20] and chemical potential[1] follow:

$$\Delta P \equiv P - P_\text{R} = -\frac{1}{2\pi^{3/2}\kappa^{-3}\beta}\frac{\epsilon - 1}{\epsilon}, \tag{S19}$$

and

$$\Delta\mu \equiv \mu - \mu_\text{R} = \frac{1}{2\beta\rho_\text{b}\kappa^{-3}\sqrt{\pi}^3}\frac{\epsilon - 1}{\epsilon} - \frac{2p^2}{3\kappa^{-3}\sqrt{\pi}}. \tag{S20}$$

Again, we demonstrate that a choice of $\kappa^{-1} = 4.5\,\text{Å}^{-1}$ is robust for these thermodynamic corrections to be highly accurate, shown in Fig. S3.

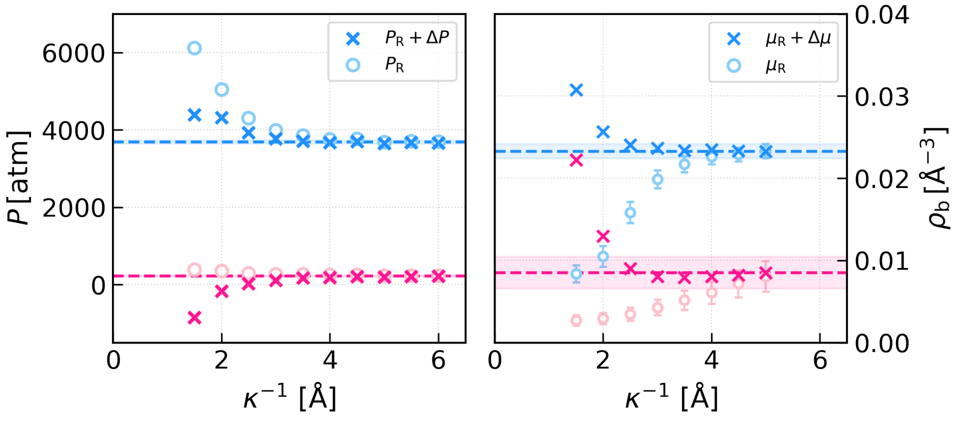

Figure S3: Consistency in bulk pressure and chemical potential between SR and LR systems. (a) The pressure of the reference SR system at various κ^{-1} without (circles) and with the long-range correction (crosses) as given by Eq. S19. The energies (at different densities) of the full LR system are indicated by the horizontal dashed lines. Results are shown representatively for for $T = 500\,\text{K}$, $\rho_\text{b} = 0.028\,\text{Å}^{-3}$ (blue) and $T = 500\,\text{K}$, $\rho_\text{b} = 0.0063\,\text{Å}^{-3}$ (pink). (b) The bulk density of the SR system with uncorrected (dots) and corrected (crosses) chemical potentials as given by Eq. S20. Horizontal lines indicate the corresponding bulk densities of the full LR system. Results are shown representatively for $T = 500\,\text{K}$, $\rho_\text{b} = 0.023\,\text{Å}^{-3}$ (blue) and $T = 500\,\text{K}$, $\rho_\text{b} = 0.0085\,\text{Å}^{-3}$ (pink).

Using the dielectric constants determined from simulations, we fit a polynomial function $\epsilon(\rho, T)$ for ease of

determining $\Delta\mu$.

S3.2. Inhomogeneous system

For inhomogeneous systems, particularly when inhomogeneities are induced by non-uniform electric fields, the effects of omitting $v_1(r)$ in the SR reference can be severe. To demonstrate that the effect of long-ranged electrostatics is readily accounted for by the restructuring potential specified in Eq. S12, we consider the response of the polar fluid under a planar sinusoidal electric field $E(z) = -\partial_z\phi(z) = E_{\rm max}\sin(2\pi z/\lambda)$. In Fig. S4, we show that the equilibrium number and charge densities of the true system are faithfully captured in the SR reference with $\phi_{\rm R}$ specified by Eq. S12. That is, we have verified that

$$\langle\hat{\rho}(\boldsymbol{r})\rangle_\phi = \langle\hat{\rho}_{\rm R}(\boldsymbol{r})\rangle_{\phi_{\rm R}}, \tag{S21}$$

$$\langle\hat{n}(\boldsymbol{r})\rangle_\phi = \langle\hat{n}_{\rm R}(\boldsymbol{r})\rangle_{\phi_{\rm R}}. \tag{S22}$$

For the polar fluid model we consider, $\hat{\rho}(\boldsymbol{r}) = \sum_{i=1}^{N}\delta(\boldsymbol{r}-\boldsymbol{r}_{\rm A,i})$ and $\hat{n}(\boldsymbol{r}) = \sum_{i=1}^{N}\sum_\alpha q_\alpha\delta(\boldsymbol{r}-\boldsymbol{r}_{\alpha,\rm i})$ with $\alpha\in\{\rm B,C\}$.

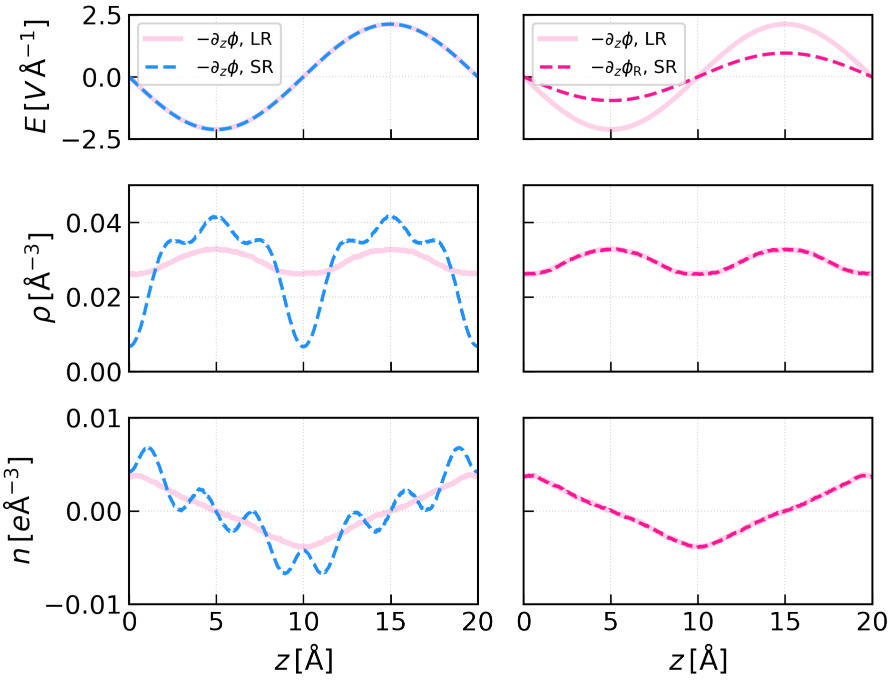

Figure S4: Restructuring potential to account for effects of LR electrostatic interaction in the reference system. **Left:** Under the same applied field $E(z) = -\partial_z\phi(z) = E_{\rm max}\sin(2\pi z/\lambda)$ with $\lambda = 20$ Å and $E_{\rm max} = -2.1$ V Å^{-1}, the SR system's number and charge density response significantly differs to that of the LR system. **Right:** Effects of LR electrostatics are readily accounted for in the SR fluid by imposing a field specified by the restructuring potential, $E(z) = -\partial_z\phi_{\rm R}(z)$. The results are from MD simulations at $T = 500$ K.

S4. GENERATION OF TRAINING DATA

Here we describe how simulation data are generated to learn $c_{\rm R}^{(1)}(\boldsymbol{r};[\varrho,\beta\varphi],T)$ and $n_{\rm R}^{(1)}(\boldsymbol{r};[\varrho,\beta\varphi],T)$ of the SR reference dipolar fluid via the neural functional method.[3] For efficient sampling of the condensed phase, training data is acquired using a combination of grand canonical Monte Carlo (GCMC) and molecular dynamics (MD) simulations,[24] with randomly generated inhomogeneous external potential energy landscapes in a planar geometry. We first use GCMC to determine the average total number of particles $N_{\rm ave}$ at a specified $\{\mu, V, T, V_{\rm ext}(z), \phi(z)\}$, and then perform canonical MD simulations with $\{N_{\rm ave}, V, T, f_{\rm ext}(z), E(z)\}$.

We have validated that this approach gives results indistinguishable from those in Ref. 15, in which we only performed GCMC simulations, yet significantly helps to converge density profiles.

Random external potential. In total, for the dipolar fluid, 2035 sets of random external controls are used for generating training data. We employ randomized simulation conditions by generating electrostatic potentials with the cosine wave form

$$\phi(z) = \frac{\phi_0}{m} \cos\left(\frac{2\pi m z}{L_z}\right), \tag{S23}$$

where the magnitude ϕ_0 is chosen uniformly in the interval $\beta q_+ \phi_0 \in [-38.2, 38.2]$ and m is an integer, which we limit to $m \in \{1, 2, 3, 4\}$. For a subset of simulations, we explicitly impose planar walls acting on the molecular center A of the form

$$V_{\mathrm{ext}}(z) = V_{\mathrm{LJ93}}(z; z_{\mathrm{lo}}) + V_{\mathrm{LJ93}}(z; z_{\mathrm{hi}}), \tag{S24}$$

where

$$V_{\mathrm{LJ93}}(z; z_*) = \varepsilon_{\mathrm{w}} \left[\frac{2}{15}\left(\frac{\sigma_{\mathrm{w}}}{|z - z_*|}\right)^9 - \left(\frac{\sigma_{\mathrm{w}}}{|z - z_*|}\right)^3\right] + \varepsilon_{\mathrm{w}} \left[\frac{2}{15}\left(\frac{\sigma_{\mathrm{w}}}{z_{\mathrm{min}}}\right)^9 - \left(\frac{\sigma_{\mathrm{w}}}{z_{\mathrm{min}}}\right)^3\right], \tag{S25}$$

where $z_{\mathrm{min}} = (2/5)^{1/6}\sigma_{\mathrm{w}}$, with $z_{\mathrm{lo}} < z < z_{\mathrm{hi}}$ located within the primary simulation cell. For these simulations, we use $\sigma_{\mathrm{w}} = 1$ Å, $\varepsilon_{\mathrm{w}} = k_{\mathrm{B}}T$, one wall is fixed at $z_{\mathrm{lo}} = 0.0$ Å and the other wall is at variable distance uniformly drawn from $z_{\mathrm{hi}} \in [2.5, 18.0]$ Å. For a subset of the simulations, the temperature is fixed at $T = 500$ K and the chemical potential is randomly chosen in the range $\mu/k_{\mathrm{B}} \in [-5000, -1000]$ K. The other subset have variable temperature uniformly distributed in the range $T \in [250, 500]$ K and chemical potential $\mu/k_{\mathrm{B}} \in [-4500, -1500]$ K. The thermal wavelengths are $\Lambda = aT^{1/2}$, where a is set to be $a = 1\,\text{Å}\,\text{K}^{-1/2}$. Since we will not consider free energy differences between different temperatures, we also set $\zeta = 1$ Å.

The procedure is repeated similarly for SPC/E water. While sampling liquid water at room temperature within the grand canonical ensemble is challenging, we can still train a neural functional with data in the range $T \in [500, 900]$ K to probe the liquid–vapor binodal behavior ($T_{\mathrm{c}} = 638.6$ K for SPC/E[25]). The number density is centered at the oxygen site.

GCMC simulations. GCMC simulations of the short-ranged system were performed with our own code `https://github.com/annatbui/GCMC`, with the standard insertion, deletion, displacement and rotation moves. The random external potentials employed are specified above. Cubic simulation boxes of length $L_x = L_y = L_z = 20$ Å with periodic boundary conditions were used. Each system was equilibrated for at least 1×10^6 MC steps. For each simulation, around 1×10^9 MC steps were attempted.

MD simulations. For each simulation with random external potentials, we then use the average number density obtained from GCMC to perform MD simulations in the NVT ensemble. MD simulations of the short-ranged system were performed with the LAMMPS simulation package.[26] Source code to implement the pair potential with short-ranged electrostatics can be accessed at `https://github.com/uccasco/LMFT`. To reduce error due to translating between ensembles, simulation boxes with larger lateral dimensions are used with $L_z = 20$ Å and $L_x = L_y = 80$ Å. Dynamics were propagated using the velocity Verlet algorithm with a time-step of 1 fs. The temperature was maintained using a Nosé–Hoover thermostat.[27,28] The molecule is evolved as a rigid body.[29] To sample the same potential as in GCMC, a spatially-varying electric field $E(z) = -\partial_z \phi(z)$ is applied. In cases where there are confining walls, `fix wall/lj93` is used to add a force on the molecular center $f(z) = -\partial_z V_{\mathrm{ext}}(z)$. The system was "equilibrated" (note that the initial configuration is from an equilibrium GCMC simulation) for 50 ps, and production runs were performed for at least 2 ns. For SPC/E water, the geometries of water molecules were constrained using the `RATTLE` algorithm.[30]

Sampling observables. For each simulation at a given set of $\{\mu, V, T, V_{\mathrm{ext}}(z), \phi(z)\}$, the number density profile

$$\rho_{\mathrm{R}}(z) = \left\langle \sum_{i=1}^{N} \delta(z - z_i^*) \right\rangle, \tag{S26}$$

where z^* denotes the coordinate of the molecular center and charge density profile

$$n_\mathrm{R}(z) = \left\langle \sum_{i=1}^{N} \sum_{\alpha} q_\alpha \delta(z - z_{\alpha,\mathrm{i}}) \right\rangle, \tag{S27}$$

with α denotes all charged sites in the molecule, were sampled with a grid-spacing $\Delta z = 0.02$ Å. The one-body direct correlation profiles are calculated from

$$c_\mathrm{R}^{(1)}(z) = \ln\left(\Lambda^3 \zeta^{-1} \rho_\mathrm{R}(z)\right) + \beta V_\mathrm{ext}(z) - \beta\mu, \tag{S28}$$

resulting from rearranging S5. In this way, we build up a dataset for both mappings,

$$\{\rho(z), \beta\phi(z), T\} \rightarrow c_\mathrm{R}^{(1)}(z),$$

and

$$\{\rho(z), \beta\phi(z), T\} \rightarrow n_\mathrm{R}(z),$$

The total computation time for the generation of the entire dataset is in the order of $\sim 10^5$ CPU hours.

S5. TRAINING

For each fluid, we train two neural networks, one to represent $c_\mathrm{R}^{(1)}([\varrho, \beta\varphi], T)$ and one for $n_\mathrm{R}^{(1)}([\varrho, \beta\varphi], T)$ following the local learning strategy.[3] The machine learning routine was implemented in Keras/Tensorflow[31] with the standard Adam optimizer.[32] The architecture of the models is shown in Figs. S5 and S6.

For both models, the input layers take in both $\rho_\mathrm{R}(z)$ and $\beta\phi(z)$, in a window of size 10 Å centered around the location of interest. With a spatial discretization of $\Delta z = 0.02$ Å, each input profile contributes 501 input nodes. To effectively learn spatial variations, the model internally computes the gradient of $\beta\phi(z)$ using a central difference scheme. Interior points are computed via symmetric finite differences, while forward and backward differences are applied at the boundaries to maintain consistency. In addition to these spatially varying inputs, a separate input node encodes the temperature T as a scalar. Each of the three input channels [$\rho_\mathrm{R}(z)$, $\beta\phi(z)$ and T] is processed independently. The density profile $\rho_\mathrm{R}(z)$ passes through a fully connected layer with 256 nodes using a softplus activation function and L2 regularization. The electrostatic potential $\beta\phi(z)$ and its gradient are independently processed through dense layers with 32 and 512 nodes, respectively, before being concatenated and further transformed by an additional 256-node layer. The temperature input is processed separately via a fully connected layer with 64 nodes. The encoded outputs from these three pathways are then concatenated into a combined feature vector, which is subsequently passed through two additional fully-connected layers, each containing 512 nodes with softplus activation and L2 regularization. The final output is a predicted profile, either $c_\mathrm{R}^{(1)}$ or $n_\mathrm{R}^{(1)}$, at position z. We note that other possible approaches have been proposed for learning the short-ranged correlations.[33–35]

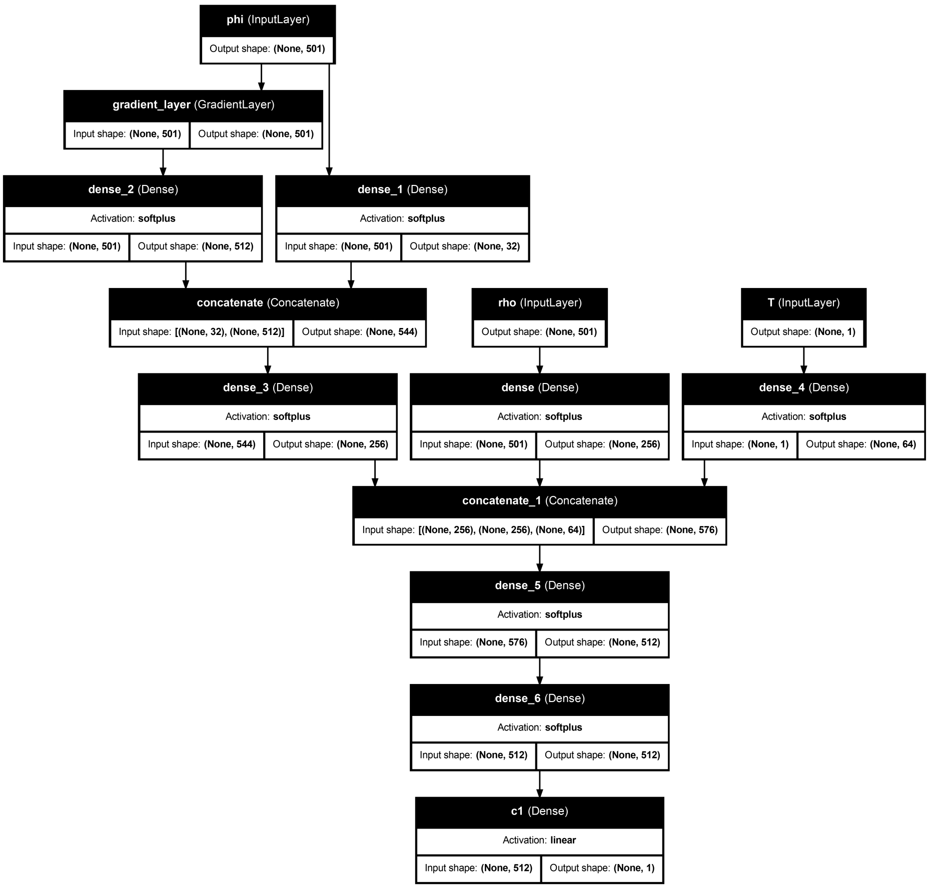

Figure S5: Architecture of the neural network for the one-body direct correlation functional $c_R^{(1)}(z;[\varrho,\beta\varphi],T)$. The output shapes indicate the variable batch size (None) and the number of nodes for each layer.

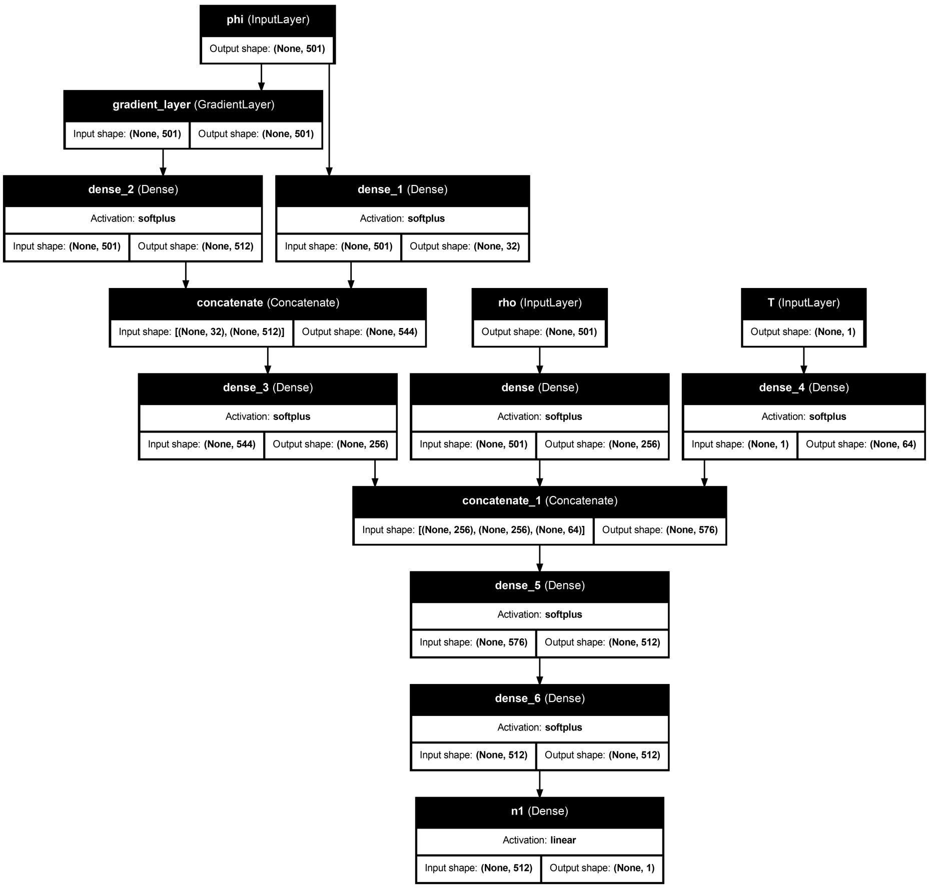

Figure S6: Architecture of the neural network for the one-body charge density functional $n_\mathrm{R}^{(1)}(z;[\varrho,\beta\varphi],T)$. The output shapes indicate the variable batch size (None) and the number of nodes for each layer.

When training the networks, we separate the 2035 independent simulations into 1278 for a training set, 410 for a validation set, and 347 for a test set. Each model was trained for 200 epochs in batch sizes of 256, and the learning rate was decreased exponentially by 5% per epoch from an initial value of 0.001. This results in a mean average error for $c_\mathrm{R}^{(1)}(z;[\varrho,\beta\varphi],T)$ of 0.0776 and for $n_\mathrm{R}^{(1)}(z;[\varrho,\beta\varphi],T)$ of $0.0002629\,e\,\text{Å}^{-3}$, which is of the same order as the estimated average noise of the simulation data for the respective profiles.

The training of the neural networks was done on a GPU (`NVIDIA GeForce RTX 3060`) in a few hours. Evaluating the trained neural functional is fast ($\sim$ milliseconds) and can be performed on a single CPU core or a GPU.

S6. USING THE NEURAL FUNCTIONALS

Inhomogeneous profiles. The central advantage of the theory concerns the determination of the one-body inhomogeneous equilibrium density and charge profiles efficiently for a given $\{\mu, V, T, V_\mathrm{ext}(z), \phi(z)\}$ by solving the Euler–Lagrange equation (Eq. S5) for $\rho(z)$ and evaluating the hyperfunctional (Eq. S6) for $n(z)$ self-consistently. Eq. S5 is solved iteratively with mixed Picard iteration.[3] The restructuring potential in a planar geometry can be recast in reciprocal space[14] as

$$\phi_\mathrm{R}(z) = \phi(z) + \frac{1}{L_z}\sum_{k\neq 0}\frac{4\pi}{k^2}\tilde{n}(k)\exp(ikz)\exp\left(-\frac{k^2}{4\kappa^2}\right), \tag{S29}$$

where $\tilde{n}$ denotes a Fourier component of n. In instances where $c_\mathrm{R}^{(1)}(z;[\rho,\beta\phi],T)$ or $n_\mathrm{R}^{(1)}(z;[\rho,\beta\phi],T)$ need to be evaluated, the neural representations learned in the previous section are used. Each calculation takes less than a minute, outperforming atomistic simulations by orders of magnitude in computational cost. This allows for a highly accurate and efficient determination of the polar fluid's response to electric field gradients of

different field strengths and wavelengths, under different thermodynamic conditions (i.e., chemical potentials and temperatures).

Phase behavior. To determine the liquid–vapor coexistence line at zero electric field, we calculate isotherms of the chemical potential as a function of the bulk density ρ_{b} from

$$\beta\mu = \ln(\Lambda^3\rho_{\mathrm{b}}) - c^{(1)}([\rho_{\mathrm{b}}, \beta\phi = 0], T), \tag{S30}$$

and perform a Maxwell construction[36] to find the coexisting liquid and vapor densities at the binodal. Such a procedure also gives the spinodal points. However, as this procedure involves supplying a bulk homogeneous density (ρ_{b}), which can be unstable in the coexistence region, it is only possible to obtain a van der Waals loop from the neural functional technique[2] at zero field. For inhomogeneous systems, i.e, as a result of an applied non-uniform electric field, we set the chemical potential and temperature and find the inhomogeneous solutions by solving the Euler–Lagrange equation (Eq. S5). The isotherms of the chemical potential, now as a function of the mean density, $\overline{\rho} = L^{-1}\int_0^L \mathrm{d}z\, \rho(z)$ where L is the total length of the domain over which $\rho(z)$ is defined, now have distinct jumps when the system undergoes phase separation. To distinguish stable solutions from metastable solutions, we also calculate the grand potential difference with respect to zero density at a fixed $\beta\phi$

$$\Delta\Omega_{\phi} = \mathcal{F}^{(\mathrm{id})}_{\mathrm{intr}}([\rho], T) + \mathcal{F}^{(\mathrm{ex})}_{\mathrm{intr}}([\rho, \beta\phi], T) - \mu \int \mathrm{d}\boldsymbol{r}\, \rho(\boldsymbol{r}) \tag{S31}$$

where the excess free energy term can be evaluated via functional line integration[35,37]

$$\beta\mathcal{F}^{(\mathrm{ex})}_{\mathrm{intr}}([\rho, \beta\phi], T) = -\int_0^{\rho} \mathcal{D}\varrho\, c^{(1)}(z; [\varrho, \beta\phi], T). \tag{S32}$$

Performing this procedure for $\beta\phi = 0$ gives results consistent with those obtained by Maxwell construction. Note that the pressure of the bulk fluid at zero field is given by $-PV = \beta\Delta\Omega_0$.

Capillary condensation. To map out the adsorption isotherms presented in the main paper, we solve, for each μ and $E(z)$ applied, the Euler–Lagrange equation to determine $\rho(z)$ and, hence, the mean number density in the slit, $\overline{\rho} = H^{-1}\int_0^L \mathrm{d}z\, \rho(z)$, where H is the slit height. To map out hysteresis loops, metastable states can be obtained with different initial guesses when solving Eq. S5.

S7. ADDITIONAL RESULTS THAT SUPPORT THE MAIN ARTICLE

S7.1. Bulk equations of state and liquid–vapor coexistence with no external electric field

The bulk equations of state under zero field for the SR and LR systems are shown in Fig. S7. In both cases, predictions from the theory are in excellent agreement with pressures from MD simulations of the bulk fluid. For subcritical temperatures, the theory predicts a van der Waals loop, as also seen when neural functional techniques are applied to the Lennard–Jones fluid.[2]

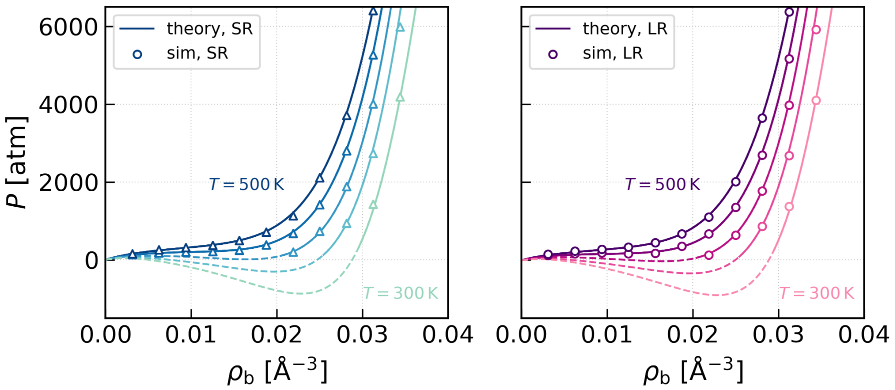

Figure S7: Bulk equation of state under zero field. Predictions from theory (lines) are in excellent agreement with explicit simulations (triangles/ circles) both for (a) the SR reference system, and (b) the LR system. The dashed lines indicate subcritcical regions where phase separation occurs. The temperatures shown include $T = 300, 400, 450$, and $500\,\mathrm{K}$.

The isotherms of the chemical potential as a function of bulk density for both the SR and LR systems are shown in Fig. S8. We also show the resulting binodal and spinodal obtained by Maxwell construction. To fit the binodal, we use the standard scaling law for the critical exponent according to the Ising universality class,[38]

$$(\rho_l(T) - \rho_v(T))^{3.06} = D\left(1 - \frac{T}{T_c}\right), \tag{S33}$$

where ρ_l and ρ_v are the densities of the coexisting phases, and D is a fitting parameter. The critical density ρ_c can then be determined from the law of rectilinear diameters[39]

$$(\rho_l(T) - \rho_v(T))/2 = \rho_c + s_2(T - T_c). \tag{S34}$$

This gives a result of $T_c \approx 431\,\mathrm{K}$ and $\rho_c \approx 0.011\,\text{Å}^{-3}$ for the SR system and $T_c \approx 445\,\mathrm{K}$ and $\rho_c \approx 0.010\,\text{Å}^{-3}$ for the LR system. This is in line with the expectation that short-ranged fluids have a lower critical temperature than their long-ranged counterparts.[40]

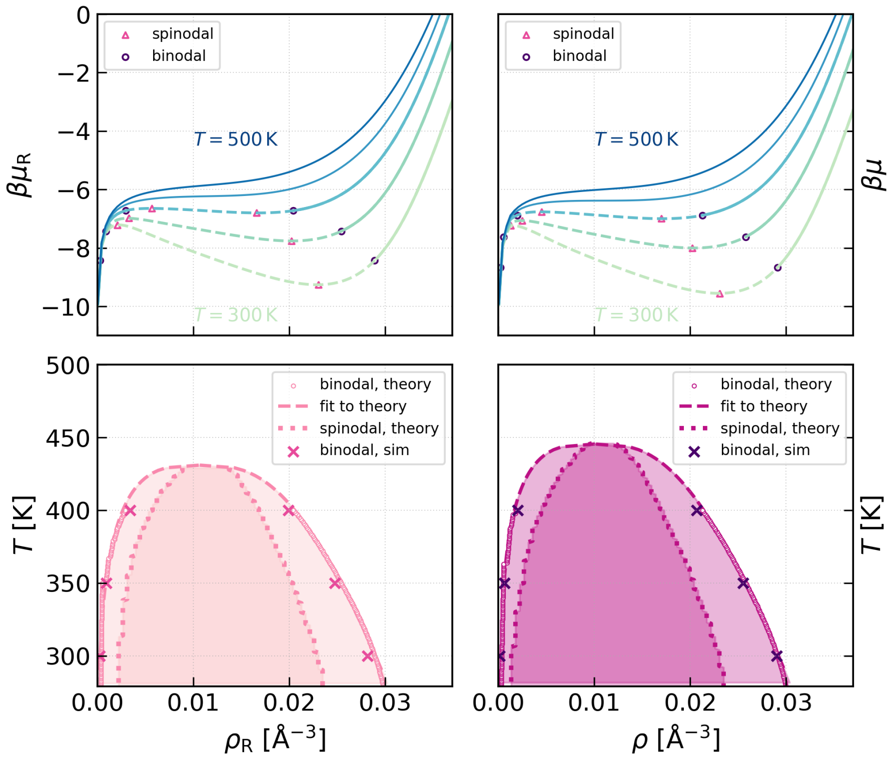

Figure S8: Liquid–vapor coexistence under zero field. (top) The chemical potential as a function of bulk density predicted from the theory for the SR (left) and LR (right) systems, shown with spinodal and binodal points obtained from Maxwell construction. The temperatures shown include $T = 300, 400, 450,$ and $500\,\mathrm{K}$. (bottom) The binodals for both the SR (left) and LR (right) systems are in very good agreement with data from direct coexistence MD simulations.

S7.2. Bulk response and phase behavior under spatially-varying external electric fields

In Fig. S9, we show that the equilibrium structure of the bulk LR polar fluid in the presence of a sinusoidal external electric field. The results from the theory are in excellent agreement with average structures obtained from equilibrium simulations.

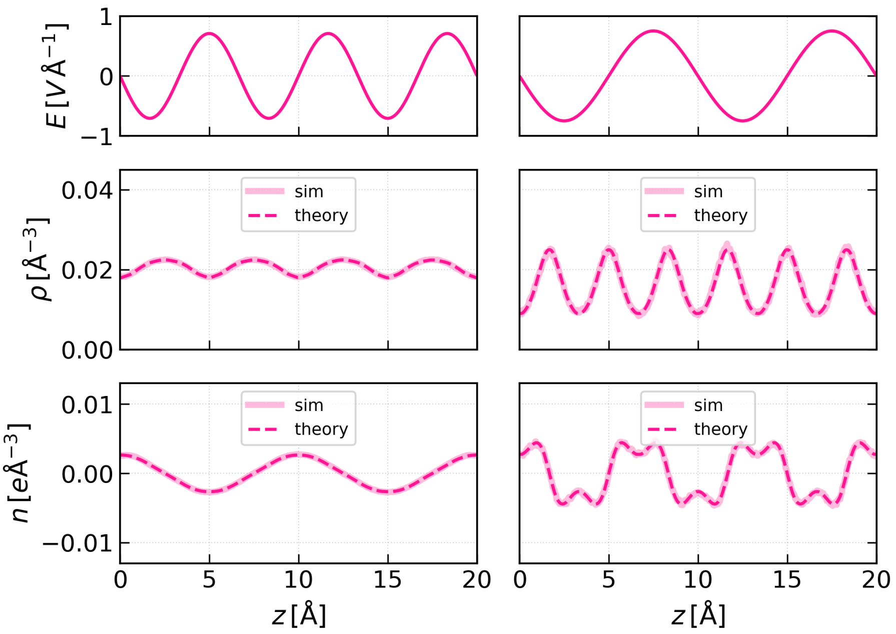

Figure S9: Electromechanical response in a polar fluid. For the LR system under sinusoidal electric field (top row), the predictions from theory for both the number (middle row) and charge densities (bottom row) are in excellent with simulations, shown representatively at $T = 500\,\text{K}$.

Fig. S10 shows the intermediate steps in obtaining the liquid-vapor binodal of the LR system that is presented in the main article. At an isotherm where phase separation is occurring, as the chemical potential μ is varied continuously, by solving the Euler-Lagrange equation, we obtain $\rho(z)$ of high density (liquid state) and of low density (vapor state), as shown for 300 K in the top row. In the middle row of Fig. S10, we present μ vs $\bar{\rho}$ obtained from the theory, both in the absence of an external electric field (left) and in the presence of an EFG (right). The bottom row in Fig. S10 shows $-\Delta\Omega_\phi$ vs $\bar{\rho}$ obtained by functional line integration (see Eq. S31).

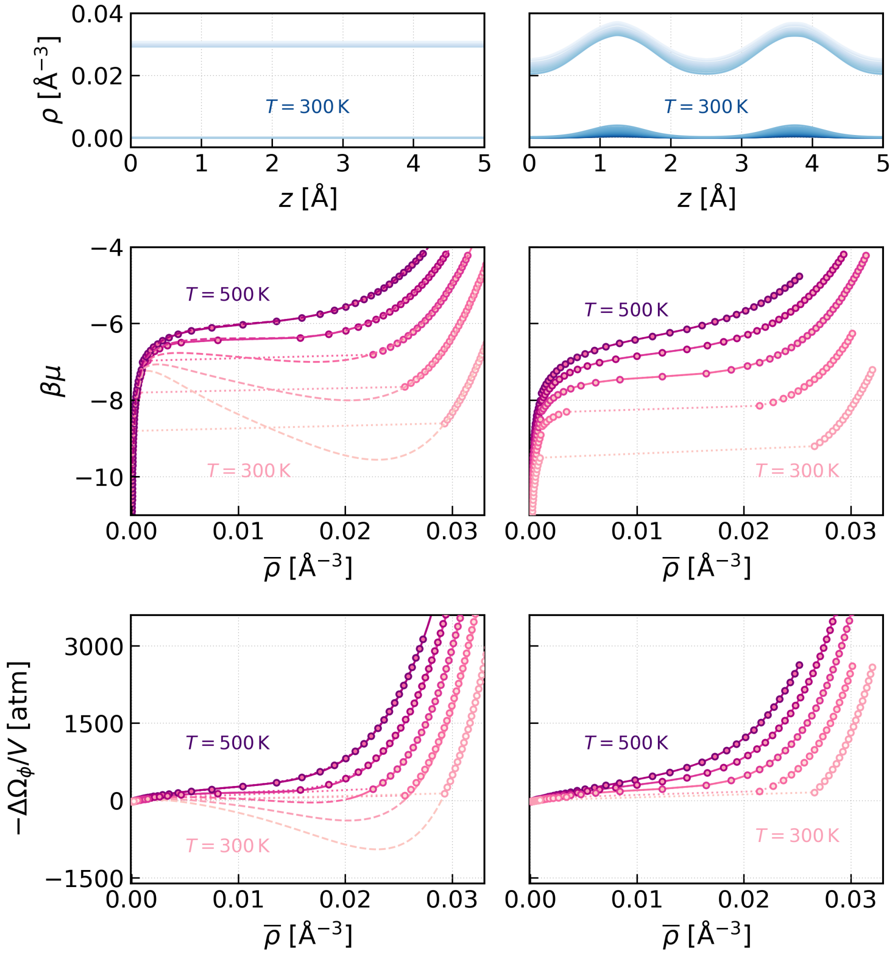

Figure S10: Liquid–vapor equilibrium under EFGs. The results are shown for a bulk system under (left) zero electric field and (right) a sinusodal electric field with amplitude $E_0 = 0.4\,\mathrm{V\,Å^{-1}}$ and wavelength $\lambda = 5\,\mathrm{Å}$. In the top row, the density profiles obtained from solving the Euler–Lagrange equation at 300 K are shown when μ is varied continuously (shown by the continuous blue shade), indicating distinct vapor–liquid phase transitions in the homogeneous fluid at zero field or inhomogenous fluid under an EFG. In the top row, we show the chemical potential isotherms obtained from solving the Euler–Lagrange equation (dots) — which in the case of zero field map directly on to the Maxwell construction approach. In the bottom row, we show the grand potential energy difference defined by Eq. S31. The temperatures shown include $T = 300, 400, 450$, and 500 K.

Figure S11 shows μ vs $\bar{\rho}$ for different combinations of $E_{\max}$ (the amplitude of the external field) and λ (the wavelength of the external field). From these curves, we map out the variation of the critical temperature with $E_{\max}$ and λ, as shown in the lower panel of Fig. S11. For the range of $E_{\max}$ and λ explored, the critical temperature decreases with increasing $E_{\max}$, and increases with increasing λ.

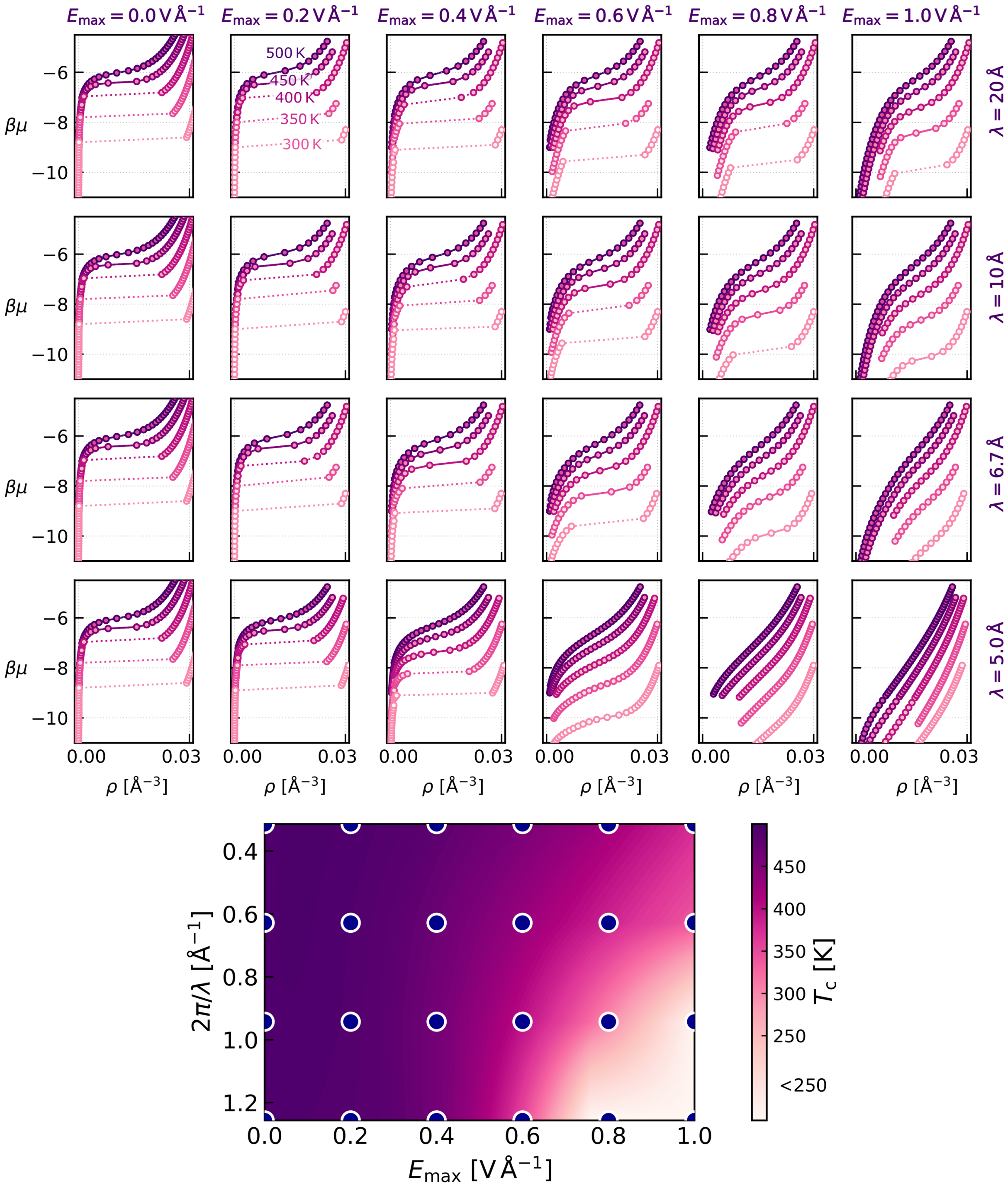

Figure S11: Fine-tuning phase transition with EFGs. The top panel shows the chemical potential isotherms at $T = 300, 350, 400, 450$ and $500\,K$ as a function of mean density at different combinations of the wavelength λ and amplitude E_{max} of the sinusoidal external electric field. In the bottom panel, the results are summarized by the critical temperature $T_c(\lambda, E_{max})$, which decreases with decreasing λ or increasing E_{max}. Circles indicate the combinations of (λ, E_{max}) shown in the top panel.

In combination with the phase behavior, the structural reorganization of the fluid can also be tuned, as demonstrated in Fig. S12.

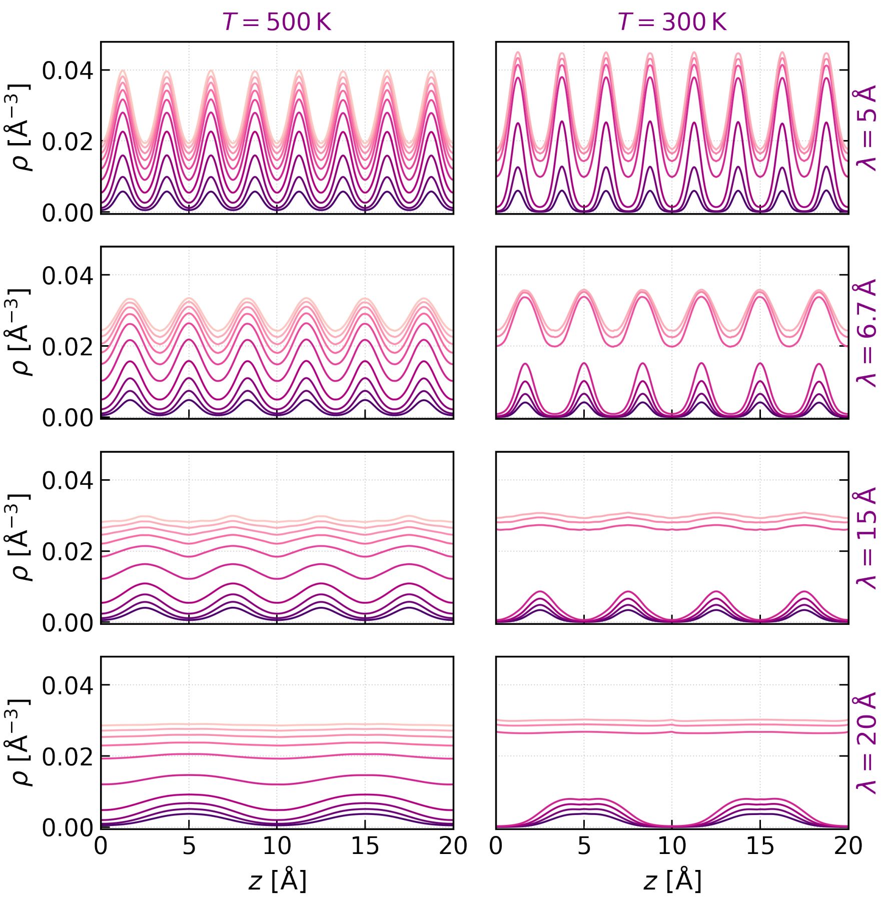

Figure S12: Fine-tuning structural reorganization with EFGs. The structural response due to dielectrophoretic rise as the wavelength λ of the applied sinusoidal electric field is changed at fixed amplitude $E_{\mathrm{max}} = 0.6\,\mathrm{V\,Å^{-1}}$ is shown (left) for $T = 500\,\mathrm{K} > T_{\mathrm{c}}(0,0)$ and (right) for $T = 300\,\mathrm{K} < T_{\mathrm{c}}(0,0)$. The chemical potential is varied continuously (shown by the color gradient) in range $\beta\mu \in [-8,-3]$ (left) and $\beta\mu \in [-11,-8]$ (right).

S7.3. Water

In Fig. S13, we show the structural reorganization due to dielectrophoretic rise in supercritical SPC/E water. Aside from the asymmetry due to the molecular structure, the dependence on field intensity and wavelength remains qualitatively similar to that of a dipolar fluid (c.f. Fig. 1 of the main article).

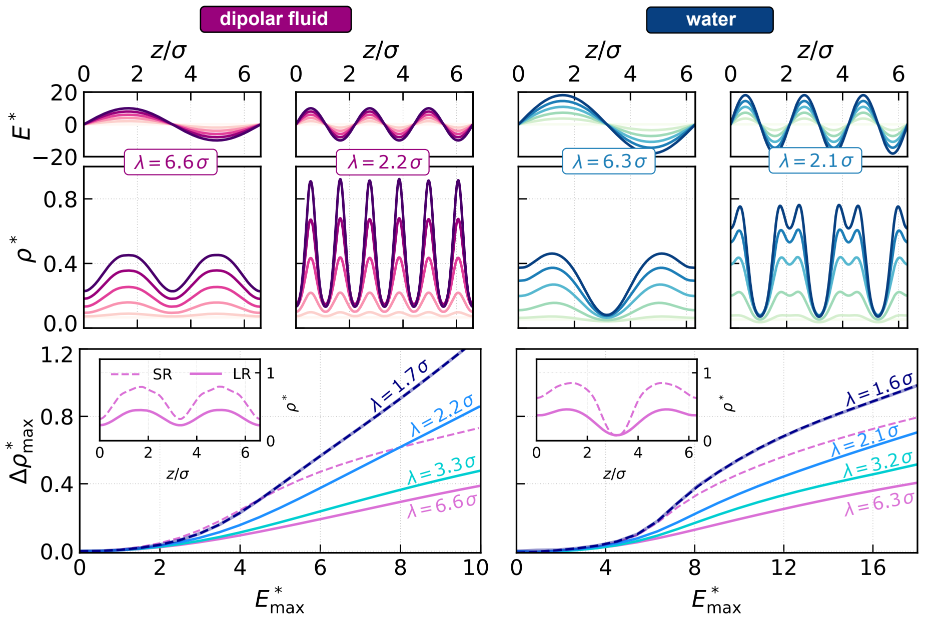

Figure S13: Dielectrophoretic rise in water under supercritical condition. The left panel shows the density response of the simple dipolar fluid (500 K, same data as in main manuscript) while the the right panel shows that for SPC/E water (700 K).

We next probe the liquid–vapor binodal. Fig. S14 shows that the critical temperature T_c shifts downward in the presence of electric field gradients, consistent with the dielectrophoretic coupling observed in simple dipolar fluids.

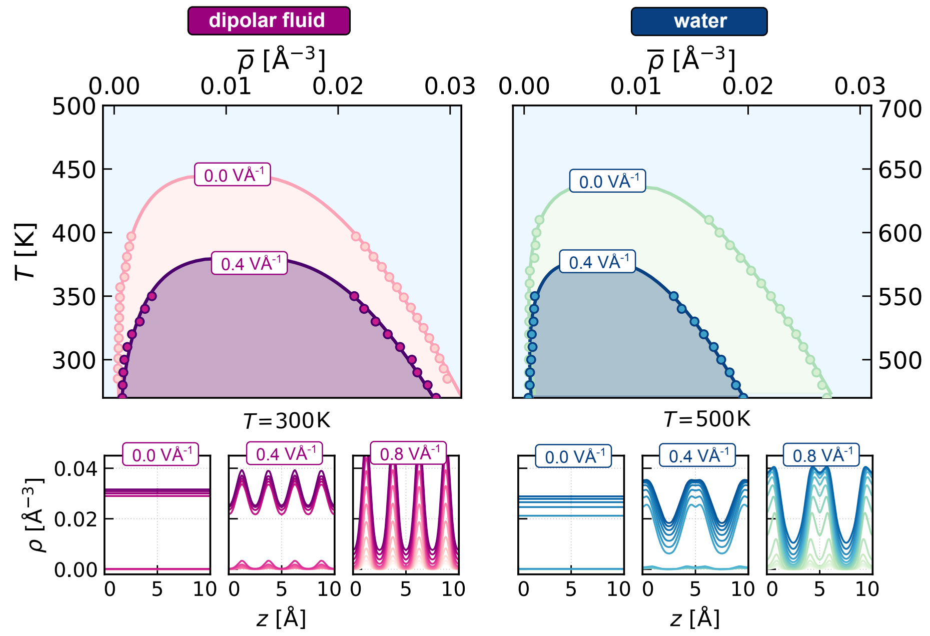

Figure S14: Water liquid–vapor equilibrium under EFGs. The intensity of the field E_{max} is labeled. The bottom panel shows the density response as the chemical potential is varied continuously.

S7.4. Interdigitated electrode simulations

Here we describe the simulation details corresponding to the snapshot in Fig. 1 of the main text. The simulated systems consist of 620 water molecules symmetrically confined between two solid substrates. Each

substrate is composed of 2048 atoms arranged on a simple cubic lattice (32×8×8, lattice parameter 2.5 Å). The orthorhombic simulation box has lateral dimensions 80 × 20 × 100 $Å^3$.

All simulations were performed with the LAMMPS package.[26] Water–water interactions were described using the SPC/E model,[19] with molecular geometry constrained via the RATTLE algorithm.[30] Substrate atoms were held rigid at their lattice positions. Water oxygen atoms interact with substrate atoms through a 12–6 Lennard–Jones potential with $\epsilon_{\rm wf} = 0.065\,{\rm kJ\,mol^{-1}}$ and $\sigma_{\rm wf} = 3.094\,Å$, with all Lennard–Jones interactions truncated and shifted at 10 Å. Electrostatic interactions were evaluated in real space up to 10 Å, with long-ranged interactions treated using PPPM Ewald summation,[41] such that the RMS force error was 10^5 smaller than the Coulomb force between two unit charges at 10 Å separation.[42]

Simulations were conducted in the canonical (NVT) ensemble at $T = 300\,$K, controlled using a Nosé–Hoover chain with 5 thermostats and a damping constant of 0.1 ps. Dynamics were propagated using the velocity Verlet algorithm with time-step of 1 fs. We performed both fixed-charge and constant-potential simulations. Constant-potential simulations employed the fluctuating-charge method,[43–45] in which the electrode atom charges are solved self-consistently at each timestep using the ELECTRODE package,[46] using Gaussian charge of width 0.554 Å. Figure S15 shows the resulting water density distributions before and after charging the electrodes, for both fixed-charge and constant-potential conditions. We observed analogous behavior in simulations comprising 310 molecules of the simple dipolar fluid at a temperature of 500 K.

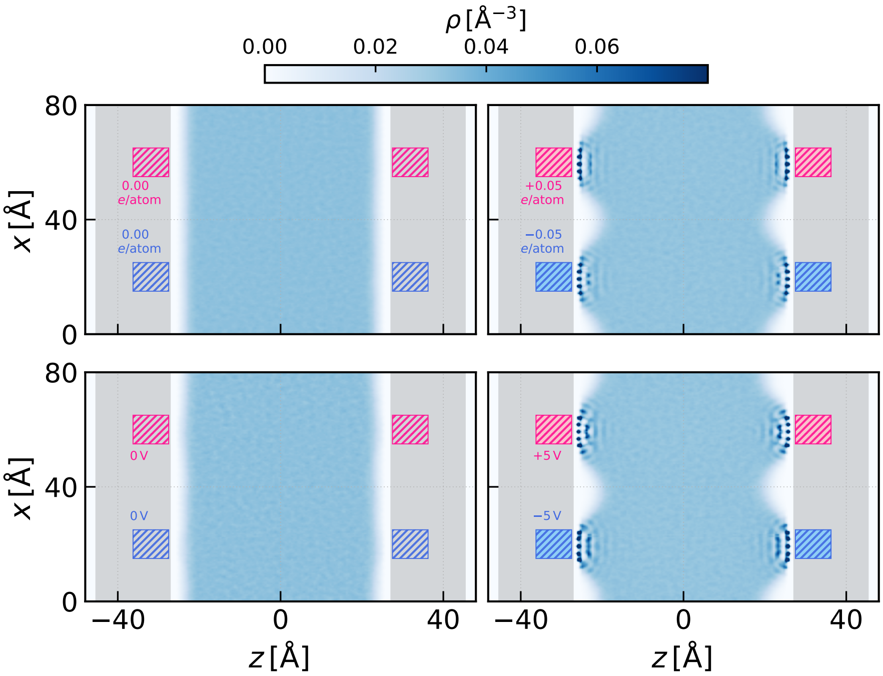

Figure S15: Density profiles of water confined between nanoscale interdigitated electrodes. For both fixed-charge (top) and constant-potential (bottom) simulations, imposing a potential difference of 10 V or a surface charge of ±0.05 e/atom enhances wetting at the walls and induces strong lateral density oscillations.

We can understand the form of the electrostatic potential arising from these substrates by approximating their inhomogeneous charge distribution as being confined to two planes at $z = \pm z_{\rm s}$, where z is along the surface normal. Denoting the direction parallel to the surfaces as x, the electrostatic potential from one of these substrates is a linear combination of functions of the form $\sin(k_n x)\exp(-k_n|z-z_{\rm s}|)$, where $k_n = 2\pi n/L_x$ ($n = 1, 3\ldots$), where L_x is the period along x. Owing to current practical limitations of the neural functional techniques that we employ, we have focused on response to electrostatic potentials with planar symmetry, i.e., either $\sin(k_n x)$ or $\exp(-k_n z)$. Far from the surface, the potential will be determined by the asymptotic form,

$$\phi_{\rm single}(x, z; z_{\rm s}) \sim \phi_0 \sin\left(\frac{2\pi x}{L_x}\right) \exp\left(-\frac{2\pi|z - z_{\rm s}|}{L_x}\right) \tag{S35}$$

For our simulation set up, the electrostatic potential midway between the substrates will be well-approximated

by $\phi(x,z;-z_s)+\phi(x,z;z_s)$.

As discussed in the main text, for L_x larger than a few molecular diameters, response to these planar potentials can be considered to report on averages over a thin slice of either $\Delta z \ll L_x$ or $\Delta x \ll L_x$. To verify the extent to which this holds, we first consider the simple polar fluid. In the central panel of Fig. S16, we compare results from: (i) the average density along z for a slice of thickness $\Delta x = 10\,\text{Å}$ centered above the negatively charged patch (depicted by the black rectangle in the leftmost panel); and (ii) the average density along z from a simulation with planar inhomogeneous symmetry, where the electrostatic potential is approximated by $\phi(z) = \phi_0[\exp(-2\pi|z+z_s|/L_x) + \exp(-2\pi|z-z_s|/L_x)]$, and where the overall density is chosen to be the same as that of the thin slice in case (i). As can be seen in the central panel, aside from a slight discrepancy far from the surface, the two sets of simulations agree well with each other. The rightmost panel shows results for a similar procedure, this time taking a thin slice centered at $z=0$ (i.e., far from the surfaces) and with $\Delta z = 10\,\text{Å}$, as depicted by the pink rectangle in the leftmost panel. In this case, the planar inhomogeneous simulation was performed with $\phi(x) = \phi_0 \sin(2\pi x/L_x)$. As can be seen in the rightmost panel, the two sets of simulations give near identical results.

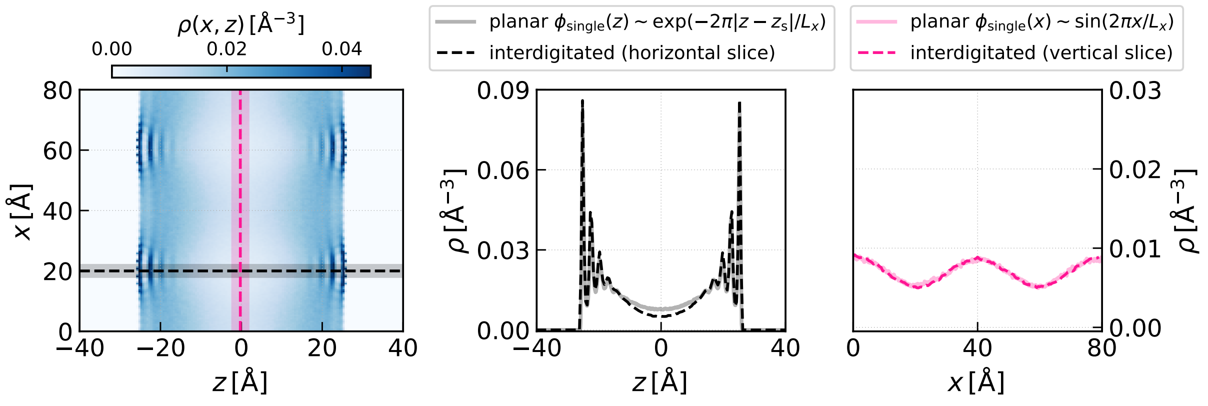

Figure S16: Response of a dipolar fluid to electric field gradients. Left: density profile of a supercritical dipolar fluid at $T = 500\,\text{K}$ confined between interdigitated electrodes. Middle/right: density responses in simulations with (middle) an exponentially decaying potential or (right) a sinusoidal potential. The cross-sections highlighted in the left panel map closely onto the corresponding reduced-potential simulations.

In Fig. S17 we show an analogous set of results obtained for water. For the simulations where we compare the exponentially decaying potentials along z (i.e., the central panel in Fig. S17), we again see good agreement between the two sets of simulations. For the sinusoidal potentials (i.e., the rightmost panel in Fig. S17), the response to the planar inhomogeneous potentials have a pronounced asymmetry that is largely suppressed in the simulation with interdigitated electrodes. This difference in geometries indicates a correlation between water's response in the x and z directions that we cannot model directly within the current limitations of the neural functional approach. Nonetheless, the most salient aspects of electromechanical response are captured and, if anything, enhanced in the full simulation with interdigitated electrodes.

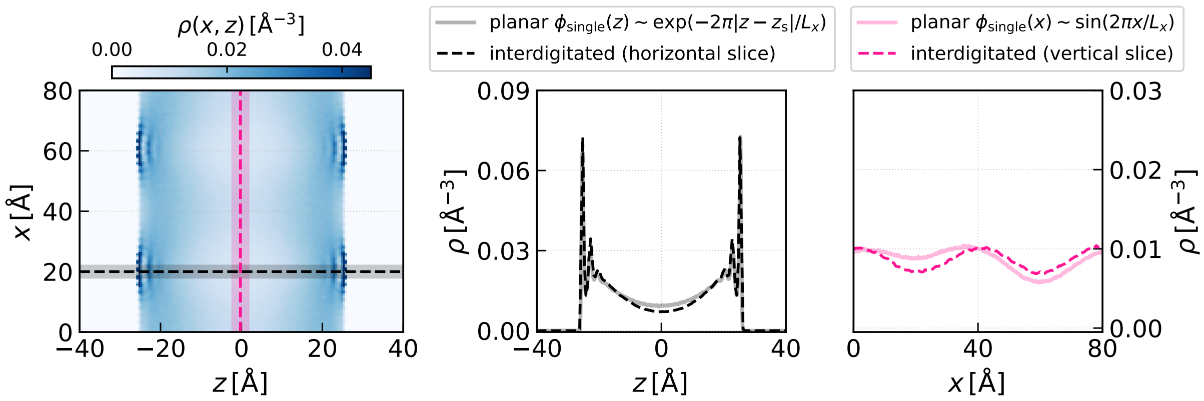

Figure S17: Response of a water to electric field gradients. Left: density profile of a supercritical SPC/E water at $T = 700\,\text{K}$ confined between interdigitated electrodes. Middle/right: density responses in simulations with (middle) an exponentially decaying potential or (right) a sinusoidal potential.

REFERENCES

[1] A. T. Bui and S. J. Cox, "A first-principles approach to electromechanics in liquids," J. Phys.: Condens. Matter **37**, 285101 (2025).

[2] F. Sammüller, M. Schmidt, and R. Evans, "Neural density functional theory of liquid-gas phase coexistence," Phys. Rev. X **15**, 011013 (2025).

[3] F. Sammüller, S. Hermann, D. de las Heras, and M. Schmidt, "Neural functional theory for inhomogeneous fluids: Fundamentals and applications," Proc. Natl. Acad. Sci. U.S.A **120**, e2312484120 (2023).

[4] R. Evans, "The nature of the liquid-vapour interface and other topics in the statistical mechanics of non-uniform, classical fluids," Adv. Phys **28**, 143–200 (1979).

[5] J. Hansen and I. McDonald, *Theory of Simple Liquids: with Applications to Soft Matter* (Elsevier Science, 2013).

[6] F. Sammüller, S. Robitschko, S. Hermann, and M. Schmidt, "Hyperdensity functional theory of soft matter," Phys. Rev. Lett. **133**, 098201 (2024).

[7] J. M. Rodgers and J. D. Weeks, "Local molecular field theory for the treatment of electrostatics," J. Phys. Condens. Matter **20**, 494206 (2008).

[8] J. M. Rodgers and J. D. Weeks, "Interplay of local hydrogen-bonding and long-ranged dipolar forces in simulations of confined water," Proc. Natl. Acad. Sci. U.S.A **105**, 19136–19141 (2008).

[9] J. D. Weeks, K. Katsov, and K. Vollmayr, "Roles of repulsive and attractive forces in determining the structure of nonuniform liquids: Generalized mean field theory," Phys. Rev. Lett. **81**, 4400–4403 (1998).

[10] J. M. Rodgers, C. Kaur, Y.-G. Chen, and J. D. Weeks, "Attraction between like-charged walls: Short-ranged simulations using local molecular field theory," Phys. Rev. Lett. **97**, 097801 (2006).

[11] Y.-g. Chen, C. Kaur, and J. D. Weeks, "Connecting systems with short and long ranged interactions: Local molecular field theory for ionic fluids," J. Phys. Chem. B **108**, 19874–19884 (2004).

[12] R. C. Remsing, S. Liu, and J. D. Weeks, "Long-ranged contributions to solvation free energies from theory and short-ranged models," Proc. Natl. Acad. Sci. U.S.A **113**, 2819–2826 (2016).

[13] A. Gao, R. C. Remsing, and J. D. Weeks, "Short solvent model for ion correlations and hydrophobic association," Proc. Natl. Acad. Sci. U.S.A **117**, 1293–1302 (2020).

[14] S. J. Cox, "Dielectric response with short-ranged electrostatics," Proc. Natl. Acad. Sci. U.S.A **117**, 19746–19752 (2020).

[15] A. T. Bui and S. J. Cox, "Learning classical density functionals for ionic fluids," Phys. Rev. Lett. **134**, 148001 (2025).

[16] W. H. Stockmayer, "Second virial coefficients of polar gases," J. Chem. Phys **9**, 398–402 (1941).

[17] E. L. Pollock and B. J. Alder, "Static dielectric properties of Stockmayer fluids," Physica A Stat. Mech. Appl. **102**, 1–21 (1980).

[18] D. Borgis, D. Laage, L. Belloni, and G. Jeanmairet, "Dielectric response of confined water films from a classical density functional theory perspective," Chem. Sci. **14**, 11141–11150 (2023).

[19] H. J. C. Berendsen, J. R. Grigera, and T. P. Straatsma, "The missing term in effective pair potentials," J. Phys. Chem. **91**, 6269–6271 (1987).

[20] J. M. Rodgers and J. D. Weeks, "Accurate thermodynamics for short-ranged truncations of Coulomb interactions in site-site molecular models," J. Chem. Phys **131**, 244108 (2009).

[21] F. H. Stillinger and R. Lovett, "Ion-pair theory of concentrated electrolytes. I. Basic concepts," J. Chem. Phys **48**, 3858–3868 (1968).

[22] J. G. Kirkwood, "The dielectric polarization of polar liquids," J. Chem. Phys **7**, 911–919 (1939).

[23] H. Fröhlich, *Theory of Dielectrics: Dielectric Constant and Dielectric Loss* (Clarendon Press, 1958).

[24] D. Frenkel and B. Smit, *Understanding Molecular Simulation: From Algorithms to Applications* (Elsevier Science, 2023).

[25] C. Vega and J. L. F. Abascal, "Simulating water with rigid non-polarizable models: a general perspective," Phys. Chem. Chem. Phys **13**, 19663–19688 (2011).

[26]A. P. Thompson, H. M. Aktulga, R. Berger, D. S. Bolintineanu, W. M. Brown, P. S. Crozier, P. J. in 't Veld, A. Kohlmeyer, S. G. Moore, T. D. Nguyen, R. Shan, M. J. Stevens, J. Tranchida, C. Trott, and S. J. Plimpton, "LAMMPS - a flexible simulation tool for particle-based materials modeling at the atomic, meso, and continuum scales," Comput. Phys. Commun **271**, 108171 (2022).
[27]W. Shinoda, M. Shiga, and M. Mikami, "Rapid estimation of elastic constants by molecular dynamics simulation under constant stress," Phys. Rev. B **69**, 134103 (2004).
[28]M. E. Tuckerman, J. Alejandre, R. López-Rendón, A. L. Jochim, and G. J. Martyna, "A Liouville-operator derived measure-preserving integrator for molecular dynamics simulations in the isothermal–isobaric ensemble," J. Phys. A: Math. Gen. **39**, 5629–5651 (2006).
[29]T. F. Miller III, M. Eleftheriou, P. Pattnaik, A. Ndirango, D. Newns, and G. J. Martyna, "Symplectic quaternion scheme for biophysical molecular dynamics," J. Chem. Phys **116**, 8649–8659 (2002).
[30]H. C. Andersen, "Rattle: A "velocity" version of the shake algorithm for molecular dynamics calculations," J. Comput. Phys **52**, 24–34 (1983).
[31]F. Chollet, *Deep Learning with Python* (Manning Publications, 2017).
[32]D. P. Kingma and J. Ba, "Adam: A method for stochastic optimization," (2017), arXiv:1412.6980.
[33]J. Dijkman, M. Dijkstra, R. van Roij, M. Welling, J.-W. van de Meent, and B. Ensing, "Learning neural free-energy functionals with pair-correlation matching," Phys. Rev. Lett. **134**, 056103 (2025).
[34]F. Sammüller and M. Schmidt, "Neural density functionals: Local learning and pair-correlation matching," Phys. Rev. E **110**, L032601 (2024).
[35]F. Sammüller and M. Schmidt, "Why hyperdensity functionals describe any equilibrium observable," J. Phys. Condens. Matter **37**, 083001 (2024).
[36]K. Binder, B. J. Block, P. Virnau, and A. Tröster, "Beyond the van der Waals loop: What can be learned from simulating Lennard-Jones fluids inside the region of phase coexistence," Am. J. Phys. **80**, 1099–1109 (2012).
[37]R. Evans, *Fundamentals of inhomogeneous fluids*, edited by D. Henderson (Dekker, New York, 1992) Chap. 3.
[38]A. M. Ferrenberg, J. Xu, and D. P. Landau, "Pushing the limits of Monte Carlo simulations for the three-dimensional Ising model," Phys. Rev. E **97**, 043301 (2018).
[39]B. Widom, "Equation of state in the neighborhood of the critical point," J. Chem. Phys **43**, 3898–3905 (1965).
[40]J. K. Johnson, J. A. Zollweg, and K. E. Gubbins, "The Lennard-Jones equation of state revisited," Mol. Phys. **78**, 591–618 (1993).
[41]R. Hockney and J. Eastwood, *Computer Simulation Using Particles* (Adam-Hilger, 1988).
[42]J. Kolafa and J. W. Perram, "Cutoff errors in the Ewald summation formulae for point charge systems," Mol. Simul. **9**, 351–368 (1992).
[43]J. I. Siepmann and M. Sprik, "Influence of surface topology and electrostatic potential on water/electrode systems," J. Comput. Phys **102**, 511–524 (1995).
[44]S. K. Reed, O. J. Lanning, and P. A. Madden, "Electrochemical interface between an ionic liquid and a model metallic electrode," J. Comput. Phys **126**, 084704 (2007).
[45]L. Scalfi, D. T. Limmer, A. Coretti, S. Bonella, P. A. Madden, M. Salanne, and B. Rotenberg, "Charge fluctuations from molecular simulations in the constant-potential ensemble," Phys. Chem. Chem. Phys **22**, 10480–10489 (2020).
[46]L. J. V. Ahrens-Iwers, M. Janssen, S. R. Tee, and R. H. Meißner, "Electrode: An electrochemistry package for atomistic simulations," J. Chem. Phys **157**, 084801 (2022).
